# Automated algorithms combining structure and function outperform general ophthalmologists in diagnosing glaucoma

**DOI:** 10.1371/journal.pone.0207784

**Published:** 2018-12-05

**Authors:** Leonardo Seidi Shigueoka, José Paulo Cabral de Vasconcellos, Rui Barroso Schimiti, Alexandre Soares Castro Reis, Gabriel Ozeas de Oliveira, Edson Satoshi Gomi, Jayme Augusto Rocha Vianna, Renato Dichetti dos Reis Lisboa, Felipe Andrade Medeiros, Vital Paulino Costa

**Affiliations:** 1 Glaucoma Service, Department of Ophthalmology, University of Campinas, Campinas, São Paulo, Brazil; 2 Department of Computer Engineering, Polytechnic School, University of São Paulo, São Paulo, São Paulo, Brazil; 3 Department of Ophthalmology and Visual Sciences, Dalhousie University, Halifax, Nova Scotia, Canada; 4 Duke University Eye Center, Department of Ophthalmology, Duke University School of Medicine, Durham, North Carolina, United States of America; Texas A&M University, UNITED STATES

## Abstract

**Purpose:**

To test the ability of machine learning classifiers (MLCs) using optical coherence tomography (OCT) and standard automated perimetry (SAP) parameters to discriminate between healthy and glaucomatous individuals, and to compare it to the diagnostic ability of the combined structure-function index (CSFI), general ophthalmologists and glaucoma specialists.

**Design:**

Cross-sectional prospective study.

**Methods:**

Fifty eight eyes of 58 patients with early to moderate glaucoma (median value of the mean deviation = −3.44 dB; interquartile range, -6.0 to -2.4 dB) and 66 eyes of 66 healthy individuals underwent OCT and SAP tests. The diagnostic accuracy (area under the ROC curve—AUC) of 10 MLCs was compared to those obtained with the CSFI, 3 general ophthalmologists and 3 glaucoma specialists exposed to the same OCT and SAP data.

**Results:**

The AUCs obtained with MLCs ranged from 0.805 (Classification Tree) to 0.931 (Radial Basis Function Network, RBF). The sensitivity at 90% specificity ranged from 51.6% (Classification Tree) to 82.8% (Bagging, Multilayer Perceptron and Support Vector Machine Gaussian). The CSFI had a sensitivity of 79.3% at 90% specificity, and the highest AUC (0.948). General ophthalmologists and glaucoma specialists’ grading had sensitivities of 66.2% and 83.8% at 90% specificity, and AUCs of 0.879 and 0.921, respectively. RBF (the best MLC), the CSFI, and glaucoma specialists showed significantly higher AUCs than that obtained by general ophthalmologists (P<0.05). However, there were no significant differences between the AUCs obtained by RBF, the CSFI, and glaucoma specialists (P>0.25).

**Conclusion:**

Our findings suggest that both MLCs and the CSFI can be helpful in clinical practice and effectively improve glaucoma diagnosis in the primary eye care setting, when there is no glaucoma specialist available.

## Introduction

Primary open-angle glaucoma (POAG) is a chronic and progressive optic neuropathy characterized by retinal nerve fiber layer thickness (RNFLT) loss and neuroretinal rim tissue thinning with progressive visual field (VF) damage [1,2]. Since early detection is essential to prevent visual impairment and blindness [3], efforts have been made to develop methods that allow clinicians to identify mild to moderate disease with adequate sensitivity and specificity.

Spectral-domain optical coherence tomography (OCT) can provide objective measurement of structural parameters of the optic nerve head and RNFLT [4,5], while standard automated perimetry (SAP) is the most widely used method to measure visual function in glaucoma patients [6]. Structural and functional damage coexist in glaucoma, and clinicians tend to interpret both sources of data when managing glaucoma suspects or patients with glaucoma [2,7]. However, structural and functional damage may not occur at the same time during the natural history of glaucoma. In early glaucoma, VF defects identified by SAP are commonly preceded by retinal ganglion cell (RGC) loss [8], whereas in advanced stages of glaucoma, OCT imaging is less likely to detect change, while further functional loss may occur [9,10]. Disagreement between structural and functional tests for glaucoma may be a result of variability, different algorithms, measurement scales and distinct characteristics of imaging and visual function testing [10,11]. Hence, the combination of structural and functional assessment is expected to improve the diagnostic accuracy of glaucoma. Several approaches have been developed to allow the combination of such parameters.

Computer-aided diagnosis (CAD) and automated computer diagnosis (ACD) have become one of the most important research topics in medical imaging and ophthalmology [12–14]. While CAD allows clinicians to use the computer output as a “second opinion” to make their final decision, ACD creates algorithms that will alone suggest a diagnosis. Supervised machine learning classifiers (MLCs) use an algorithm which learns from a training dataset with labeled categories. Furthermore, the function generated by the algorithm maps the new data into the existing categories which allows the prediction of cases with minimum classification error. The MLCs have been used to improve the sensitivity and specificity of glaucoma detection [15–23]. Algorithms built from MLCs enable computers to learn from a large amount of data generated by imaging methods and/or VF tests, gaining ability to discriminate between healthy and glaucomatous individuals. In a previous study, we have demonstrated that MLCs have adequate diagnostic accuracy when using both OCT and SAP parameters [23].

Another method that combines structural and functional information was described by Medeiros et al [24]. They estimated the number of RGCs based on OCT and SAP measurements [8] and calculated a combined structure—function index (CSFI), which corresponds to the percentage of RGC loss in a given eye compared to an age-matched healthy eye. The CSFI has been shown to perform better than isolated measurements of structure and function in the detection of glaucoma [24].

Despite all available technology to diagnose glaucoma, it is still unclear whether these methods surpass the diagnostic ability of glaucoma specialists and general ophthalmologists [25]. The current study was designed to test the ability of MLCs using OCT and SAP parameters to discriminate between healthy and glaucomatous individuals, and to compare it to the diagnostic ability of the CSFI, general ophthalmologists and glaucoma specialists exposed to the same OCT and SAP data.

## Methods

We conducted an observational, cross sectional and comparative study at the Glaucoma Service of the University of Campinas, Brazil. The study was approved by the University of Campinas Ethics Committee. In accordance with the Declaration of Helsinki, all participants gave written informed consent. All participants were older than 40 years with best-corrected visual acuity ≥ 0.3 logMAR, refractive error < 5.0 spherical diopters and 3.0 cylinder diopters, open angles on gonioscopy and reliable SAP and frequency-doubling technology (FDT) exams, with false-positive errors, false-negative errors and fixation losses not exceeding 15%. Participants with retinal diseases, uveitis, non-glaucomatous optic neuropathy, secondary glaucoma, advanced glaucomatous damage (defined as mean deviation [MD] ≤ -12 dB) [26], pseudophakia or aphakia, and significant cataract according to the Lens Opacification Classification System III were excluded [27].

The inclusion criteria for healthy eyes were: intraocular pressure (IOP) < 21mmHg with no history of elevated IOP, no family history of glaucoma, two consecutive, reliable and normal FDT exams (defined as no point in the pattern deviation plot with P < 5% and pattern standard deviation within 95% normal limits), and normal optic discs (with intact neuroretinal rims and no disc hemorrhages, notches, localized pallor, or cup-to-disc ratio asymmetry > 0.2). For the glaucoma group, the inclusion criteria were: two IOP measurements ≥ 21mmHg in different days, 2 consecutive and reliable FDT exams showing glaucomatous defects (defined as 2 or more adjacent points in the pattern deviation plot with P < 5% or pattern standard deviation with P < 5%), and optic nerve damage compatible with glaucoma (defined when at least 2 of the following characteristics were present: cup-disc ratio > 0.6, cup-disc ratio asymmetry > 0.2, focal defects of the neuroretinal rim, and disc hemorrhage). If both eyes were eligible, one eye was randomly selected to be included in the study.

All eligible participants underwent a complete ophthalmologic examination, including slit lamp biomicroscopy, IOP measurement using Goldmann applanation tonometry, gonioscopy, dilated fundus evaluation using a 78 diopters lens, FDT (Full-threshold program N-30, Welch Allyn, Skaneateles, NY, USA), SAP (SITA Standard 24–2, size III stimulus, Humphrey Field Analyzer II 745, Carl Zeiss Meditec Inc., Dublin, CA) and Spectral-domain OCT (Cirrus, Carl Zeiss Meditec Inc, version 5.1.1.6, Dublin, CA, USA). All OCT, SAP and FDT testing were done within 6 months. Since SAP was used as the functional parameter for the MLCs, CSFI and the analysis by ophthalmologists, the FDT was used as inclusion criteria to avoid selection bias and an artificial increase of SAP diagnostic accuracy. All OCT images were acquired with dilated pupils by a single, well-trained examiner (LSS). The protocol used for RNFLT measurements was the optic disc cube. This protocol places a circumpapillary circle (1.73mm radius and 10.87mm length) around the optic disc, from which the information about peripapillary RNFLT is obtained. The peripapillary circular scan had to be well centered, with a signal strength ≥ 7, and no motion artifact or segmentation error within the area of RNFLT analysis.

### Machine learning classifiers

In a previous study, we trained 10 MLCs using both OCT and SAP parameters to diagnose glaucoma in a population of 48 healthy individuals and 62 glaucoma patients [23]. The following algorithms were tested: Bagging (BAG), Naive-Bayes (NB), Multilayer Perceptron (MLP), Radial Basis Function Network (RBF), Random Forest (RAN), Ensemble Selection (ENS), Classification Tree J48 (CTREE), Ada Boost M1 (ADA), Support Vector Machine Linear—LibSVM Linear (SVML) and Sequential Minimal Optimization or Support Vector Machine Gaussian (SVMG) [28–37]. The classifiers were developed using data mining machine learning environment software Weka version 3.7.0 (Waikato Environment for Knowledge Analysis, The University of Waikato, New Zealand) [38] with hyperparameters set to their default values, except for SVML and SVMG. The specific hyperparameters for SVML were normalize = true and probability estimates = true, whereas for SVMG we used build logistic models = true, standardize training data and RBF Kernel. All 10 MLCs were tested with 10-fold cross validation.

In our previous study [23], MLC training sessions were supervised with all 17 OCT parameters and 3 SAP parameters (a total of 20 features). OCT parameters used for the MLCs were global peripapillary RNFLT, 4 quadrants (superior, inferior, nasal, and temporal) and 12 clock hour RNFLT measurements. All OCT data were aligned according to the orientation of the right eye. Thus, clock hour 9 of the circumpapillary scan represented the temporal side of the optic disc for both eyes. SAP parameters included in the analysis were MD, pattern standard deviation (PSD), and glaucoma hemifield test (GHT). For the GHT results, we assigned within normal limits a value of 1; borderline, 2; and outside normal limits, 3. The MLCs developed in our previous study [23] were tested in the population of the present study.

### Combined structure and function index

The CSFI was calculated for each eye according to the methods described by Medeiros et al. [24] In summary, the CSFI is calculated by subtracting the estimated number of RGCs from the expected value for an age-matched healthy eye. A weighted scale according to the severity of disease merges average estimates of RGC numbers from SAP and OCT data. The index corresponds to the percent of RGC loss reflected by the weighted scale.

### General ophthalmologists and glaucoma specialists

Three general ophthalmologists and three glaucoma specialists (fellowship-trained) with at least 5 years of practice were selected as observers. The ophthalmologists, masked to all clinical information, except data obtained from the OCT and SAP exams from the study eyes, were asked to grade each participant in: 1 (definitely normal), 2 (probably normal), 3 (undecided), 4 (probably glaucoma), or 5 (definitely glaucoma) [39]. Subsequently, a structure-function grading was obtained using a 15-point likelihood score scale, which corresponds to the sum of the scores assigned by the three observers of each group. The cumulative score was employed to determine the sensitivity and specificity of general ophthalmologists and glaucoma specialists to diagnose glaucoma.

### Statistical analysis

Continuous variables were compared using the Student’s T test and categorical variables were analyzed using the Chi-Square or the Fisher Exact test. A bootstrap resampling procedure (n = 1000 resamples) was used to derive confidence intervals. Diagnostic intraclass agreement for general and specialist observers was evaluated with kappa statistics (k). Strength of agreement was categorized according to the method proposed by Landis and Koch [40].

The ROC curves were built and sensitivities at fixed specificities of 80 and 90% were estimated for each MLC, CSFI, general ophthalmologists and glaucoma specialists. The receiver operating characteristic (ROC) curve is a graphical plot represented by the true positive rate against the false positive rate for the different possible cut-points of a diagnostic test [41]. The area under ROC curve (AUC) is used as a measure of the performance of a diagnostic test. The AUC range from 0.5 to 1.0: an area of 0.5 suggests that the diagnostic test has no discriminatory ability, whereas an area of 1.0 is considered the ideal test with perfect diagnostic accuracy. The MLC producing the largest AUC was used for comparison. Comparisons between AUCs were made using the nonparametric DeLong method [42]. P values < 0.05 were considered statistically significant. All analyses were performed using the open-source software R 3.3.2 (R Foundation for Statistical Computing, Vienna, Austria) [43].

## Results

The study population (124 eyes of 124 participants) consisted of 66 healthy and 58 POAG participants with early to moderate VF damage (median value of the MD: −3.44 dB; first quartile: -6.0; third quartile: -2.4; range: −0.14 to −11.98 dB). The population included in our previous study was different and composed of 110 eyes of 110 participants (48 healthy and 62 POAG participants) [23]. Table 1 summarizes the demographic and clinical characteristics of the participants of the current study. Both FDT and SAP showed significantly lower MD and higher PSD values in the glaucoma group. Forty-three of the glaucoma patients (74%) were classified as having early and 15 patients (26%) had moderate VF defects [26]. The median estimated numbers of RGCs in the healthy and glaucoma groups were 939,567 and 622,452, respectively (P < 0.001).

**Table 1:** Demographics and clinical characteristics of the study population.

	Healthy (N = 66)	Glaucoma (N = 58)	P value
Age (years), median (IQR)	55 (51–61.8)	60 (54–62)	0.077
Left eye, no. (%)	33 (50.0)	28 (48.3)	0.859
Female gender, no. (%)	41 (62.1)	28 (48.3)	0.148
Ethnicity (White; Black; Mixed; Asian), no. (%)	39 (59.1); 14 (21.2); 12 (18.2); 1 (1.5)	20 (34.5); 21 (24.5); 17 (29.3); 0	0.030
VA (logMAR), median (IQR)	0 (0)	0.05 (0–0.1)	0.001
SE (D), median (IQR)	0.25 (-0.25 to + 0.75)	0.25 (-0.25 to +1.5)	0.180
IOP (mmHg), median (IQR)	13.0 (11–14)	13.5 (12–14.8)	0.062
Medications, median (IQR)	0	3 (2–4)	<0.001
SAP MD (dB), median (IQR)	-0.65 (-1.6 to 0)	-3.44 (-6.0 to -2.4)	<0.001
SAP PSD (dB), median (IQR)	1.84 (1.5–2.2)	4.31 (2.8–6.0)	<0.001
FDT MD (dB), median (IQR)	-0.50 (-1.2 to 0.4)	-3.27 (-5.0 to -1.9)	<0.001
FDT PSD (dB), median (IQR)	3.87 (3.2–4.3)	5.41 (4.6–6.9)	<0.001
SAP*rgc* (x1000 cells), median (IQR)	1151 (1045–1263)	857 (688–944)	<0.001
OCT*rgc* (x1000 cells), median (IQR)	939 (845–1071)	589 (484–746)	<0.001
WRGC (x1000 cells), median (IQR)	939 (855–1070)	622 (536–753)	<0.001
CSFI (%), median (IQR)	4.5 (-4.1 to 15.4)	36.9 (27.4 to 44.8)	<0.001
Glaucoma specialist likelihood scale, median (IQR)	4 (3–5.8)	13.5 (9.3–15)	<0.001
General ophthalmologist likelihood scale, median (IQR)	4 (4–8)	12 (9–14)	<0.001

IQR = interquartile range; VA = visual acuity; SE = spherical equivalent; D = diopters; dB = decibels; SAP = standard automated perimetry; FDT = frequency doubling technology; MD = mean deviation; PSD = pattern standard deviation; SAP*rgc* = SAP-derived estimate of total number of retinal ganglion cells; WRGC = weighted number of retinal ganglion cells based on OCT and SAP measurements; OCT*rgc* = *OCT*-derived estimate of total number of retinal ganglion cells; CSFI = combined structure-function index.

**Fig 1:**
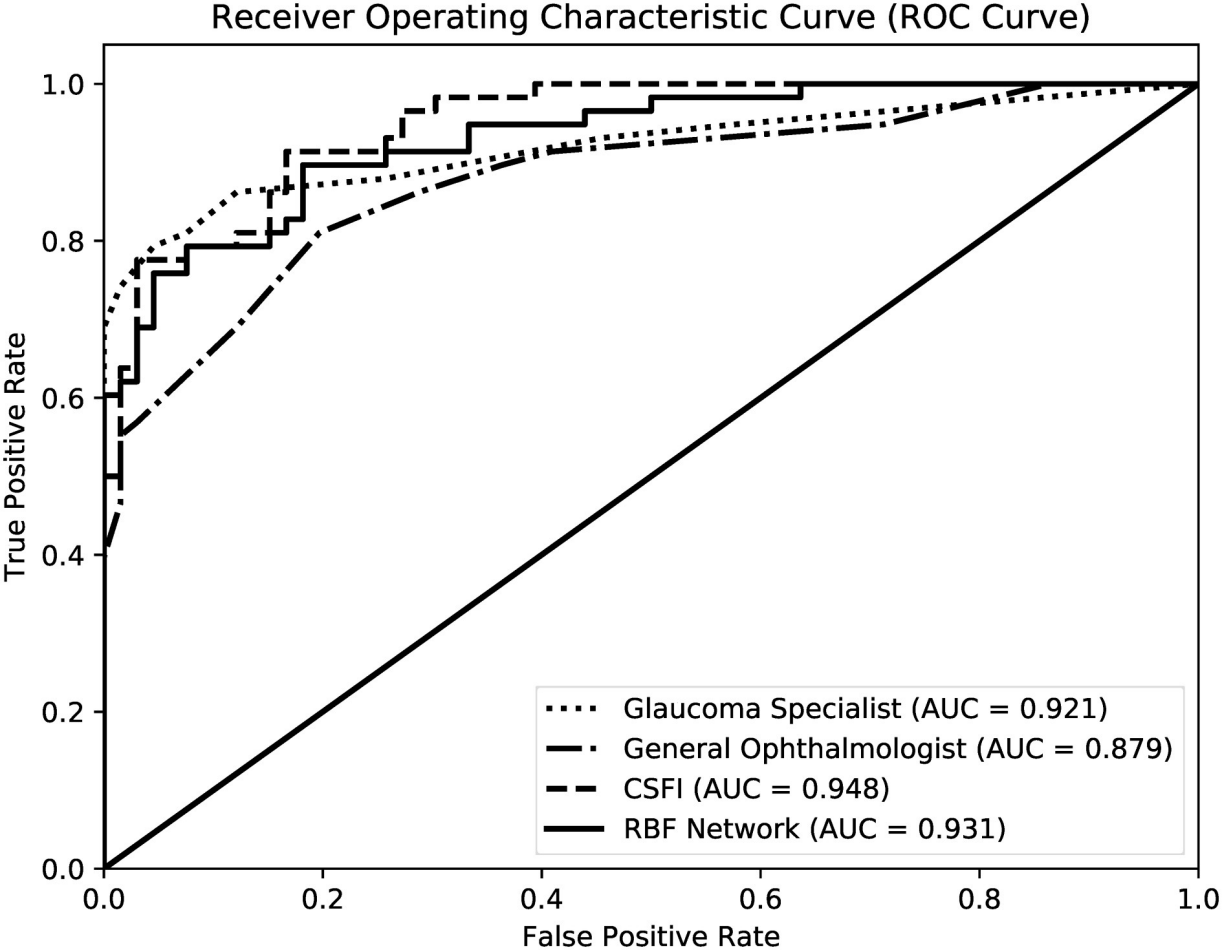
ROC Curves of the best MLC (RBF), CSFI, general ophthalmologists and glaucoma specialists.

The AUCs obtained with MLCs ranged from 0.805 (CTREE) to 0.931 (RBF). The sensitivity at fixed specificities of 80% and 90% ranged from 77.8% and 51.5% (CTREE) to 93.1% and 82.8%, respectively (MLP and BAG, Table 2). The median CSFI was 4.5% (IQR -4.1% to 15.4%) and 36.9% (27.4% to 44.8%) in the healthy and glaucoma groups, respectively (P < 0.001). The CSFI had a sensitivity of 91.4% and 79.3% at fixed specificities of 80% and 90%, respectively, and the highest AUC (0.948) when compared to the other methods (Table 2 and Fig 1).

**Table 2:** Areas under ROC curve (AUC) and sensitivities (%) at fixed specificities of 80% and 90% obtained with SD-OCT and SAP data using MLCs, CSFI, glaucoma specialists and general ophthalmologists.

	AUC	Sensitivity at 90% specificity	Sensitivity at 80% specificity
ADA	0.874	76.9%	82.7%
BAG	0.871	82.8%	93.1%
CTree	0.805	51.6%	77.8%
ENS	0.853	76.0%	83.8%
MLP	0.895	82.8%	93.1%
NB	0.923	81.0%	86.2%
RAN	0.910	81.0%	87.9%
RBF	0.931	75.9%	90.0%
SVML	0.913	80.3%	84.8%
SVMG	0.924	82.8%	89.7%
CSFI	0.948	79.3%	91.4%
Glaucoma Specialists	0.921	83.8%	87.2%
General Ophthalmologists	0.879	66.2%	81.2%

Abbreviations: ADA, Ada Boost M1; BAG, Bagging; CTREE, Classification Tree; ENS, Ensemble Selection; MLP, Multilayer Perceptron; NB, Naive-Bayes; RBF, Radial Basis Function Network; RAN, Random Forest; SVML, Support Vector Machine Linear; SVGM, Support Vector Machine Gaussian; CSFI, Combined Structure-Function Index.

The median structure-function gradings obtained from the 15-point likelihood score scale determined by the 3 general ophthalmologists were 4 (IQR 4–8) for healthy individuals and 12 (IQR 9–14) for glaucoma patients (P < 0.001). The median gradings obtained by the 3 glaucoma specialists were 4 (IQR 3–5.8) in healthy individuals and 13.5 (IQR 9.3–15) in glaucoma participants (P < 0.001). General ophthalmologists grading had sensitivities of 81.2% and 66.2% at fixed 80% and 90% specificities, respectively (AUC = 0.879). The corresponding figures for glaucoma specialists were 87.2% and 83.8%, respectively (AUC = 0.921). The kappa coefficient (k) was 0.67 (95% CI: 0.59 to 0.75) among general ophthalmologists and 0.86 (95% CI: 0.82 to 0.90) among glaucoma specialists, indicating substantial and almost perfect agreement, respectively [40].

Table 3 compares the AUCs between the 4 tested methods. RBF (the best MLC), the CSFI, and glaucoma specialists showed significantly higher AUCs than that obtained by general ophthalmologists. However, there were no significant differences between the AUCs obtained by RBF, the CSFI, and glaucoma specialists (P>0.25).

**Table 3:** Comparison of AUCs obtained with RBF, CSFI, general ophthalmologists and glaucoma specialists (P values).

	CSFI	Glaucoma Specialists	General Ophthalmologist
MLC (RBF Network)	0.309	0.648	0.046
CSFI	--	0.254	0.007
Glaucoma Specialist	--	--	0.030

Abbreviations: MLC, Machine Learning Classifier; RBF Network, Radial Basis Function Network; CSFI, Combined Structure-Function Index.

## Discussion

In order to improve the diagnostic accuracy in glaucoma, the combination of anatomical and functional data has shown to be superior than isolated structural or functional methods [44–46]. Several studies have used different MLCs combining imaging and visual field datasets to test the ability to differentiate healthy from glaucomatous eyes [16,47–51]. Recently, Kim et al. showed good performance of four MLCs prediction models (C5.0, random forest, SVM, and k-nearest neighbor) using clinical, structural and functional features (age, IOP, cornea thickness, mean RNFLT, GHT, MD and PSD), with AUCs ranging from 0.967 to 0.979 [48]. However, the authors allowed the inclusion of patients with advanced glaucomatous damage, which probably increased the accuracy of their model. In another study, relevance vector machine (RVM) and subspace mixture of Gaussian (SSMoG) models using OCT and SAP data (RVM AUC = 0.845 and SSMoG AUC = 0.869) performed significantly better than MLCs developed only with OCT data (RVM AUC = 0.809 and SSMoG AUC = 0.817) [49]. However, the performance was similar to that obtained only with SAP parameters (RVM AUC = 0.815 and SSMoG AUC = 0.841). Their study may have been somewhat biased by the use of SAP to both classify the eyes and train the MLCs. Racette et al. [50] showed that when combining relevant Heidelberg Retina Tomograph (HRT) and Short-wavelength automated perimetry (SWAP) parameters with RVM (AUC = 0.93), the discrimination between glaucomatous and non-glaucomatous eyes improved when compared to the diagnostic accuracy of RVM trained on HRT (AUC = 0.88) or SWAP (AUC = 0.76) alone. Raza et al. described a method based on cluster analysis to identify abnormal areas on OCT and SAP. The combination of OCT and SAP data improved the diagnostic accuracy (AUC = 0.868) compared to OCT (AUC = 0.818) or SAP (AUC = 0.797) alone [51]. As mentioned before, our group tested 10 supervised MLCs, combining OCT and SAP data, and the algorithm RAN showed the best performance (AUC = 0.946) for early glaucoma detection [23]. Following this study, we now tested the same MLCs in a completely different population and reported findings that are in agreement with our previous findings and the literature. The RBF Network classifier applying all 20 parameters from OCT and SAP provided the highest AUC (0.931) among the 10 MLCs. Although the RBF Network achieved a sensitivity of 75.9% at a fixed specificity of 90%, the BAG, MLP and SVMG algorithms achieved the highest sensitivity (82.8%) at a fixed specificity of 90% among all MLCs.

The results of structural and functional tests can also be merged into the CSFI [24], an estimate of the percentage of RGCs loss, compared to the expected value in age-matched healthy eyes. Harwerth et al. showed that SAP sensitivity values provide good estimates of the amount of histologically-measured RGC numbers in the retina, which was also closely related with the estimates obtained from OCT RNFLT measurements. The combination of those two estimates improves the precision of the final calculation of neuronal losses [8]. However, instead of simply averaging the two estimates, Medeiros et al. employed a weighting strategy based on MD values [24], which took into consideration differences in performance of SAP and OCT at different stages of the disease. In their study, the CSFI had an AUC of 0.94 to discriminate glaucomatous from normal eyes, which was larger than OCT RNFLT (AUC = 0.92, p = 0.008), SAP MD (AUC = 0.88, p<0.001), and SAP visual field index (AUC = 0.89, p<0.001). In our study, the diagnostic performance of the CSFI was excellent (AUC = 0.948) and comparable to those exhibited by MLCs. In addition to facilitate early glaucoma diagnosis by interpreting large and complex data, automated algorithms combining structure and function have potential practical implications for clinicians. The CSFI is useful to stage disease severity and to predict structural and functional loss [24], with an intuitive interpretation of percentage loss of neuronal tissue. On the other hand, MLCs provides an automated classification into categories (diseased or non-diseased), followed by prediction class probabilities of an accurate classification. In other words, it can provide an estimate of how accurate is the prediction given by the MLC (from 0 to 100%). Both of them will be useful in helping the ophthalmologist when facing a glaucoma suspect. A potential advantage of the CSFI is that it has recently become commercially available, which is not true for the MLCs we described.

When algorithms are proposed to enhance the diagnostic accuracy of a given test, it is important to compare their performance with the standard of care, best represented by the judgement of clinicians. Previous studies have compared the diagnostic performance of imaging techniques with general ophthalmologists and glaucoma specialists [52,53]. Vessani et al. compared the ability of subjective assessment of stereophotographies by general ophthalmologists and by one glaucoma specialist with objective measurements by OCT, confocal scanning laser ophthalmoscopy, and scanning laser polarimetry (SLP) in discriminating glaucomatous and normal eyes. The AUC obtained by general ophthalmologists (0.80) was significantly lower than those obtained by the glaucoma expert (0.92), OCT (0.92) and SLP (0.91) [52]. This finding contrasts with a report by DeLéon-Ortega et al., which showed a significantly larger AUC of glaucoma expert assessment of stereophotographies (0.90) compared to the objective measurements from OCT (0.85) and SLP (0.84) [53]. However, in their study, the reference standard was defined as optic disc damage based on the slit-lamp exam, which explains why stereophotos resulted in a larger AUC. Interestingly, only structural tests were evaluated in these studies, and the authors used an older version of OCT (time-domain). Furthermore, examiners were exposed to a different technology (stereophotography) and were not allowed to evaluate the results of the imaging tests to classify the eyes. In our series, we elected to include both structural and functional data, which is closer to what is used in clinical practice, and we chose to expose clinicians to the same data utilized by MLCs and the CSFI.

In the current study, the diagnostic ability to detect glaucoma of artificial intelligence (MLC) using structural and functional parameters was compared to the ability of the CSFI and the judgment made by ophthalmologists. We found similar performances for the best MLC, CSFI and glaucoma specialists. However, all three outperformed the general ophthalmologists’ assessment. Glaucoma specialists had a higher sensitivity (83.8%) at a fixed specificity of 90% and a larger AUC (0.921) when compared to general ophthalmologists (sensitivity of 66.2% at a fixed 90% specificity and AUC = 0.879, P = 0.03). General ophthalmologists performed worse than the best MLC (P = 0.046) and CSFI (P = 0.007, Table 3). On the other hand, glaucoma specialists had a similar diagnostic performance compared to the best MLC (P = 0.648) and CSFI (P = 0.254, Table 3). In fact, specialists provided the highest sensitivity (83.8%) at a fixed specificity of 90% among all methods analyzed. This is, to our knowledge, the first study indicating that automated methods using structural and functional data outperform general ophthalmologists in diagnosing glaucoma, suggesting that their diagnostic ability may be enhanced to a level closer to a glaucoma specialist. Recent investigations have also shown comparable, or even better performance between ACD systems based on MLCs and well-trained and experienced clinicians [54,55]. Kloppel et al. compared the ability of one MLC (Support Vector Machine—SVM) to six experienced radiologists in differentiating sporadic Alzheimer’s disease from controls. SVMs correctly classified 95% of the cases, while radiologists correctly classified the scans in 65–95% (median = 89%) [54]. Burlina et al. demonstrated the efficacy of a deep convolutional neural network combined with SVM for automated retinal image analysis and age-related macular degeneration severity categorization. The evaluation of this automated algorithm using 5664 color fundus images showed comparable diagnostic accuracy and substantial agreement for the classification when compared to ophthalmologist grading [55].

The current study has some limitations. Despite the use of IOP, clinical assessment of the optic disc and FDT to define glaucoma, there is a lack of an independent gold standard for glaucoma that is neither structural nor functional in nature. The option of creating a panel of glaucoma specialists to define normal and glaucomatous patients was avoided, since this approach would favor the diagnostic ability of the glaucoma specialists group. This explains why objective criteria based on FDT and clinical examination of the optic disc were employed to define glaucoma. The design of the study (case control) probably overestimated the diagnostic performance of all tested methods by creating two distinct populations of healthy and glaucomatous individuals [56]. Although SD-OCT was used in our study, the estimating RGCs from OCT data was based on time-domain OCT for CSFI development. It is possible that modifications would be necessary to compensate for the change in technologies. The presence of media opacities, unreliable OCT and SAP exams or imaging artifacts could also be potential sources of bias susceptible to alter data for both CSFI and MLCs. Longitudinal studies are needed to evaluate the ability of algorithms that combine structural and functional data to predict which individuals suspected of having glaucoma will show progression over time. The general ophthalmologists in this study may not represent all clinicians who are dealing with glaucoma patients in the primary care setting. Their ability to detect disease may vary depending on many factors such as experience, knowledge and available technology. We assumed that most general ophthalmologists have access to OCT and SAP printouts, which may not be the case in developing countries. Hence, the current findings may not be generalized to all general ophthalmologists. Finally, the performance of general ophthalmologists and glaucoma specialists could have been enhanced if they were exposed to sterereophotographies. However, it would not be fair to compare their ability to diagnose glaucoma with information that was not included in the tested algorithms.

In conclusion, MLCs, CSFI and glaucoma specialists performed better than general ophthalmologists using only OCT and SAP data for the detection of early to moderate glaucoma. Although our sample size was limited, which warrants further investigation with a larger population of glaucoma patients and controls, our findings suggest that both MLCs and the CSFI can be helpful in clinical practice and effectively improve glaucoma diagnosis in the primary eye care setting, when there is no glaucoma specialist available.

## Supporting information

S1 File Dataset. Dataset from study population and machine learning classifiers training and testing features.
(XML)
